# Nasal administration of mesenchymal stem cells restores cisplatin-induced cognitive impairment and brain damage in mice

**DOI:** 10.18632/oncotarget.26272

**Published:** 2018-10-30

**Authors:** Gabriel S. Chiu, Nabila Boukelmoune, Angie C.A. Chiang, Bo Peng, Vikram Rao, Charles Kingsley, Ho-Ling Liu, Annemieke Kavelaars, Shelli R. Kesler, Cobi J. Heijnen

**Affiliations:** ^1^ Neuroimmunology Laboratory, Department of Symptom Research, University of Texas MD Anderson Cancer Center, Houston, TX 77030, USA; ^2^ Department of Bioinformatics and Computational Biology, University of Texas MD Anderson Cancer Center, Houston, TX 77030, USA; ^3^ Section of Neuropsychology, Department of Neuro-Oncology, University of Texas MD Anderson Cancer Center, Houston, TX 77030, USA; ^4^ Department of Imaging Physics, University of Texas MD Anderson Cancer Center, Houston, TX 77030, USA

**Keywords:** chemobrain, mesenchymal stem cells, nasal administration, mitochondrial function, cisplatin

## Abstract

Cognitive impairments are a common side effect of chemotherapy that often persists long after treatment completion. There are no FDA-approved interventions to treat these cognitive deficits also called ‘chemobrain’. We hypothesized that nasal administration of mesenchymal stem cells (MSC) reverses chemobrain. To test this hypothesis, we used a mouse model of cognitive deficits induced by cisplatin that we recently developed. Mice were treated with two cycles of cisplatin followed by nasal administration of MSC. Cisplatin treatment induced deficits in the puzzle box, novel object/place recognition and Y-maze tests, indicating cognitive impairment. Nasal MSC treatment fully reversed these cognitive deficits in males and females. MSC also reversed the cisplatin-induced damage to cortical myelin. Resting state functional MRI and connectome analysis revealed a decrease in characteristic path length after cisplatin, while MSC treatment increased path length in cisplatin-treated mice. MSCs enter the brain but did not survive longer than 12-72 hrs, indicating that they do not replace damaged tissue. RNA-sequencing analysis identified mitochondrial oxidative phosphorylation as a top pathway activated by MSC administration to cisplatin-treated mice. Consistently, MSC treatment restored the cisplatin-induced mitochondrial dysfunction and structural abnormalities in brain synaptosomes. Nasal administration of MSC did not interfere with the peripheral anti-tumor effect of cisplatin. In conclusion, nasal administration of MSC may represent a powerful, non-invasive, and safe regenerative treatment for resolution of chemobrain.

## INTRODUCTION

Chemotherapy is still one of the most effective treatments to combat cancer. However, chemotherapy is associated with many negative side effects, including fatigue, pain, numbness and tingling in hands and feet, and cognitive impairments [1–4]. In view of the rapidly growing number of cancer survivors, there is an urgent need for an effective therapeutic intervention to treat these neurotoxicities.

Chemotherapy-induced cognitive impairment, commonly known as “chemobrain”, involves impairment in working memory, attention, processing speed, concentration, and executive function [1, 3, 5–7]. Chemobrain has been observed in 78% of cross-sectional and 69% of prospective longitudinal studies performed between 1995 and 2012 in patients treated for breast cancer [3]. Resting state functional magnetic resonance imaging (rsfMRI) analysis of patterns of brain network connectivity indicate that chemobrain is associated with abnormalities in the functional connectome indicative of inefficient information processing [5–7]. In approximately 30% of patients, symptoms of chemobrain persist long after completion of treatment thereby severely hampering quality of life and limiting their home and occupational activities [4]. There is also a growing concern that chemotherapy may increase the risk for accelerated aging and later neurodegenerative conditions [6].

Cognitive impairments also frequently develop as a result of cisplatin treatment [8, 9]. Cisplatin being the most widely used chemotherapeutic, is part of the standard treatment for numerous malignancies including head and neck, testicular, ovarian, and non-small cell lung cancer [10, 11]. Cisplatin penetrates into the brain in low concentrations [12, 13].

We recently developed a mouse model of cisplatin-induced cognitive impairment. We demonstrated that cisplatin treatment results in long lasting cognitive dysfunction in association with impaired neurogenesis, white matter damage and a loss of neuronal dendritic spines and arborizations [14, 15]. In addition, we demonstrated that deficiencies in mitochondrial bioenergetics likely underlie the cisplatin-induced cognitive dysfunction [15].

Recent advances in regenerative medicine identified MSC as a potential treatment for cerebral stroke, traumatic brain injury, hypoxic ischemic encephalopathy, status epilepticus, cranial radiation damage as well as for neurodegenerative diseases [16, 17, 26, 18–25]. We demonstrated before that nasal MSC administration to neonatal mice with ischemic cerebral damage has life-long efficacy and does not induce malignancies or other pathologic abnormalities [23, 27]. Here, we tested the hypothesis that nasal administration of MSC is an efficacious non-invasive strategy for reversing chemobrain and investigated the mechanism of repair via RNA sequencing and assessment of synaptosomal mitochondrial function. In addition, we investigated whether nasal MSC administration after completion of cisplatin treatment interfered with tumor recurrence or growth.

## RESULTS

### Effect of MSC on cisplatin-induced cognitive impairment

**Figure 1:**
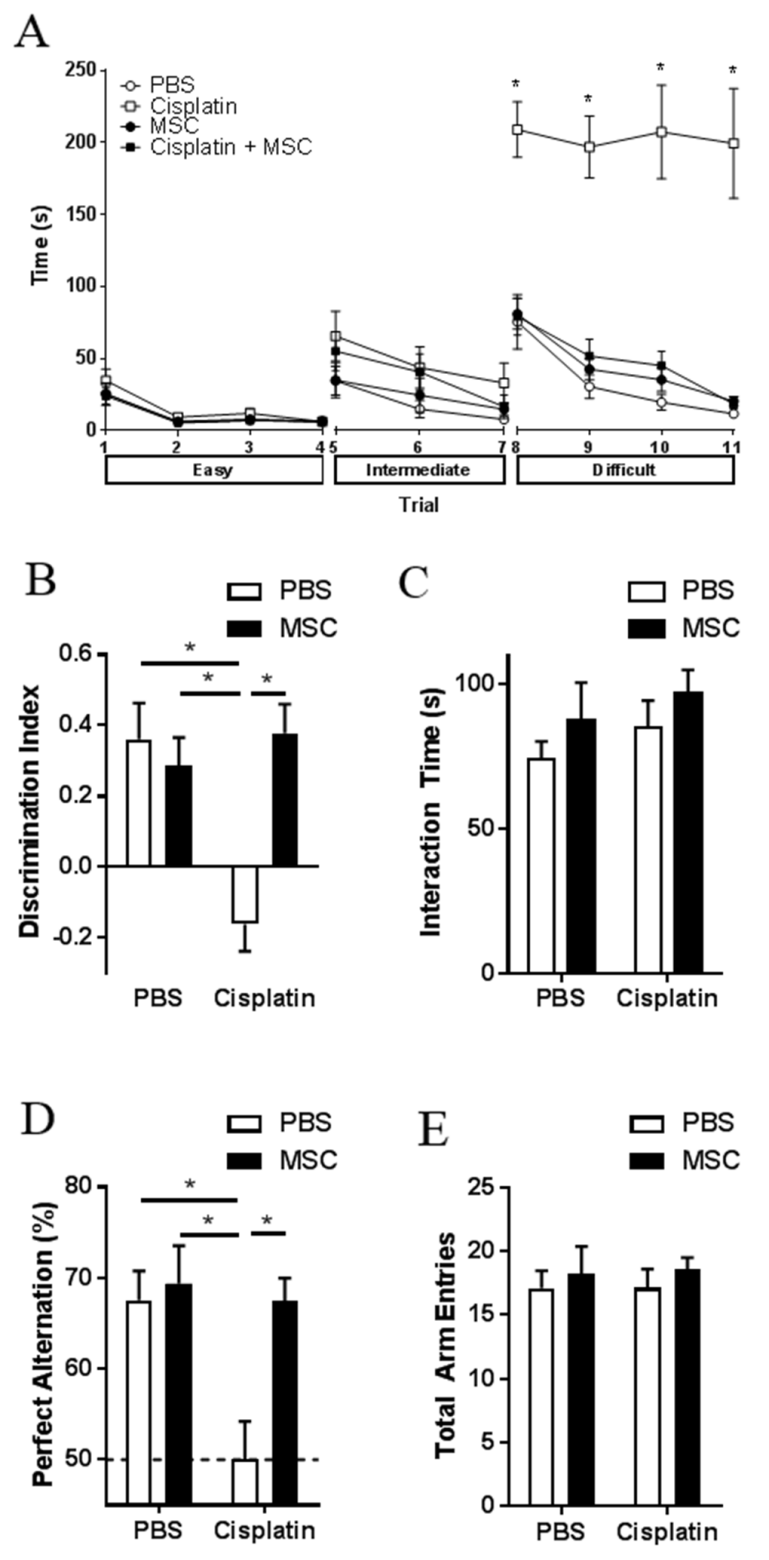
Effect of nasally administered MSC on cisplatin-induced cognitive impairments Male mice were treated with cisplatin (2.3 mg/kg/day) for two 5-day cycles with 5 days of rest in between, followed by 2 doses of 1x10^6^ MSC administered nasally at 48- and 96-h post-cisplatin. **(A)** The PBT was performed 7-10 days after the last dose of MSC. Each animal was given 3 consecutive trials per day for the first 3 days and 2 consecutive trials on day 4. The test is divided into 3 difficulties, easy (trials 1-4), intermediate (trials 5-7), and difficult (trials 8-11). During the easy trials, the animals are allowed free passage between the light and dark compartment via the connecting tunnel. In the intermediate trials, the tunnel is covered with normal bedding and animals must burrow through to reach the dark side. In the difficult trials, the tunnel is blocked by a lid and the animals must learn to remove the blockage before they can escape to the dark compartment. Time until entrance into the dark compartment is recorded. Results are expressed as mean ± SEM; n = 8 in 2 separate cohorts. *Tukey post hoc:* ^*^P < 0.05 versus PBS controls. **(B)** NOPRT was performed 4 days after the completion of the PBT. The discrimination index was calculated as (T_Novel_ – T_Familiar_)/(T_Novel_ + T_Familiar_); 0 represents no preference for the novel object. Results are expressed as means ± SEM; n = 7-8 in 2 separate cohorts. Two-way ANOVA Cisplatin x MSC interaction (F [1, 26] = 12.98, p = 0.0013), *Tukey post hoc:* ^*^P < 0.05 versus PBS controls. **(C)** Total interaction times in the NOPRT were not affected by cisplatin and MSC treatment. Two-way ANOVA Cisplatin x MSC interaction (F [1, 26] = 0.0792, p = 0.7805). **(D)** The percentage spontaneous alternation in a Y-maze was determined 1 day after the completion of the NOPRT. Dotted line indicates random chance. Results are expressed as means ± SEM; n = 7-8. Two-way ANOVA Cisplatin x MSC interaction (F [1, 26] = 4.786, p = 0.0379), *Tukey post hoc:* ^*^P < 0.05. **(E)** Total arm entries in the Y-maze were not affected by cisplatin and MSC treatment. n = 7-8. Two-way ANOVA Cisplatin x MSC interaction (F [1, 26] = 0.0135, p = 0.9083).

Male and female mice were treated with two cycles of cisplatin (2.3 mg/kg for 5 days), [15] followed by nasal administration of MSC at 48 and 96 h after the last dose of cisplatin. Behavioral testing started 7-10 days after MSC administration. We used the puzzle box test (PBT) as a measure of executive functioning [28]. The PBT consists of a total of 11 trials at 3 levels of complexity (for details see methods and legend to Figure 1A). The results in Figure 1A demonstrate that in male mice, cisplatin did not affect the time to enter the dark compartment in the easy trials 1-4 (open tunnel) and the intermediate trials 5-7 (bedding-covered tunnel). However, in the difficult trials 8 to 11 (tunnel blocked by lid), cisplatin-treated male mice took significantly more time to enter the dark compartment than control mice, indicating impaired executive function. The heatmap of trial 8 showed that the cisplatin-treated mice spent approximately 60% of the time in the area around the tunnel, indicating that the delay in entering the dark compartment was not due to lack of motivation but rather to a deficient executive function (Supplementary Figure 1A-1B). In female mice, the effect of cisplatin on performance in the puzzle box was similar to what was observed in males (Supplementary Figure 2A). Interestingly, in both male and female mice nasal administration of two doses of 1×10^6^ MSC at 48 and 96 h after completion of cisplatin treatment completely reversed the impaired executive function induced by cisplatin (Figure 1A (males) and Supplementary Figure 2A (females)).

The effect of cisplatin and MSC on spatial and working memory was tested using the novel object/place recognition test (NOPRT) that is based on the innate preference of rodents for novelty (Figure 1B–1C and Supplementary Figure 2B-2C). Cisplatin treatment decreased the preference for the novel object/place in the NOPRT, indicating impaired spatial and/or working memory (Figure 1B (males) and Supplementary Figure 2B (females)). Nasal administration of MSC normalized performance of cisplatin-treated male and female mice in the NOPRT. We observed no significant differences between groups in total interaction time with the objects between cisplatin-treated mice vs control (Figure 1C (males) and Supplementary Figure 2C (females)), indicating that the effect of cisplatin was not due to decreased interest or motivation.

Cisplatin treatment reduced the number of perfect alternations in the Y-maze test in males and females, without changes in the number of total arm entries (Figure 1D–1E (males) and Supplementary Figure 2D-2E (females)). A reduction in perfect alternations indicates a decrease in spatial memory. Consistent with what we observed in the PBT and the NOPRT, nasal MSC administration resulted in recovery from the cisplatin-induced deficit in the Y-maze in both males and females (Figure 1D and Supplementary Figure 2D). Because the effects of cisplatin and MSC on cognitive function was similar in males and females, we focused our further studies on males.

We showed earlier that this regimen of cisplatin treatment does not lead to differences in total locomotor activity in a novel environment nor in immobility in the forced swim test [15] indicating that there are no confounding effects of sickness, anxiety, or depression on performance in the cognitive tasks.

### Migration of nasally administered MSC into the brain

**Figure 2:**
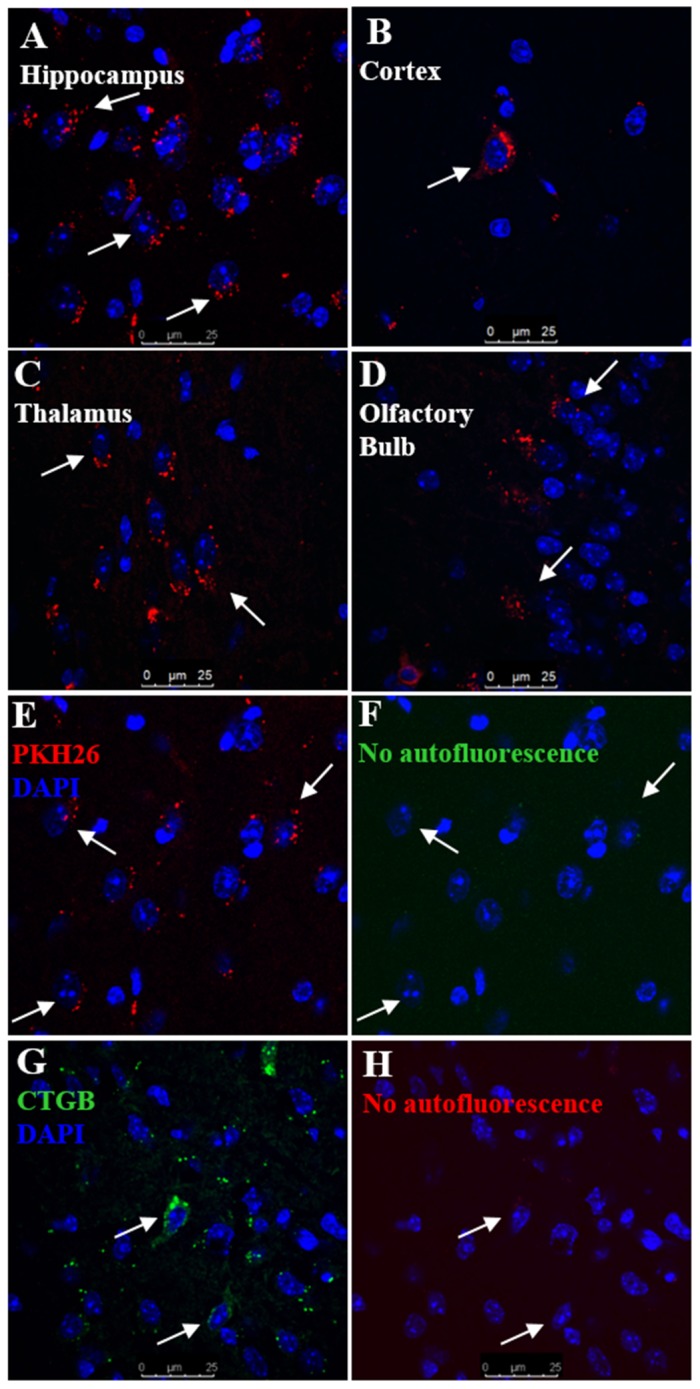
Migration of nasally administered MSC into the brain Mice were treated with cisplatin for two 5-day cycles followed by nasal administration of PKH26- or CTGB-labelled MSC at 48 h after cisplatin. Brains were taken 12 h after nasal administration of MSC and analyzed for the presence of PKH26^+^ or CTGB^+^ MSC by confocal microscopy. PKH26^+^ cells were detected in the **(A)** hippocampus, **(B)** cortex, **(C)** thalamus, and **(D)** olfactory bulb. **(E)** PKH26^+^ cells were detected in the hippocampus while **(F)** no signal was detected in the green channel. **(G)** CTGB^+^ cells were also detected in the hippocampus while **(H)** no signal was detected in the red channel. Therefore we can conclude that the fluorescent signals are specific and cannot be attributed to autofluorescence.

MSC were labelled with either PKH-26 (red) or CTGB (green). Labeled MSC were nasally administered 48 h after the last dose of cisplatin. Brains were harvested 12 h later. PKH-26^+^ MSC were detected in the hippocampus, cortex, thalamus, and the olfactory bulb (Figure 2A–2D, and Supplementary Figure 3). MSC were not detected in the hypothalamus. We did not detect any signal in the green channel, confirming that the signal was specific and cannot be attributed to autofluorescence (compare Figure 2E and 2F). Similar results were obtained with CTGB as the fluorescent tag to trace the MSC (Figure 2G) with no autofluorescence detected in the red channel (Figure 2H). In a separate set of experiments, MSC isolated from GFP-transgenic mice were administered to cisplatin-treated animals as before. To determine the fate of GFP^+^ cells, we performed qPCR analysis of the GFP encoding sequence at 12- and 72- hrs after GFP^+^ MSC administration. At 12 hours after MSC administration, the GFP transgene was detectable in the hippocampus in all mice (4 out of 4). We no longer detected the GFP transgene in any of the mice at 72 hours or 7 days after MSC administration. These findings indicate that MSC are present in the brain parenchyma at 12-hrs, but are gone by 72-hrs and did not transdifferentiate into other cell types such as neurons or glia.

### Effect of nasally administered MSC on cisplatin-induced changes in brain functional connectivity

**Figure 3:**
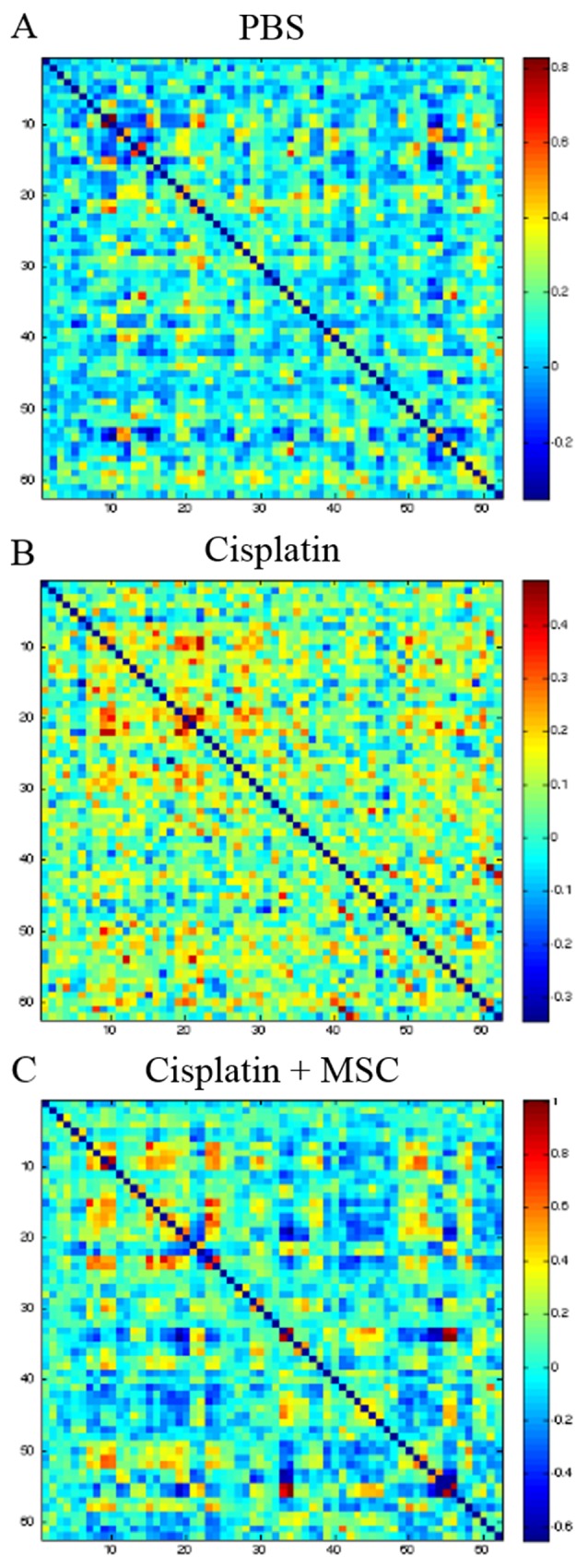
Effect of cisplatin and MSC on brain functional connectivity After completion of behavioral analysis, rsfMRI followed by connectome analysis was performed as described in the methods section. Comparing the connectome of control **(A)** and cisplatin-treated mice reveals that cisplatin treatment results in significantly lower connectome organization **(B)** which appears more “noisy” than the cisplatin + MSC connectome **(C)**.

The functional connectome is a map of connections among brain regions that provides insight into the patterns of brain network connectivity that support efficient information processing. To test whether cisplatin induces changes in the functional connectome, mice underwent rsfMRI after completion of behavioral testing (4-5 weeks after MSC administration). A 62x62 correlation matrix was calculated for each mouse using regions identified via a co-registered T2-weighed volume normalized to a C57BL/6J mouse brain template. The results in Figure 3A-3C depict the heatmaps for the correlation matrices in each group. Cisplatin-treated mice demonstrated significantly lower characteristic path-length compared to PBS mice (F = 16.4, p = 0.02, Figure 3). Regionally, cisplatin-treated mice showed significantly lower nodal clustering of left thalamus, left striatum, left cortical subplate, left pallidum and right vermis (all p < 0.05, Table 1).

**Table 1:** Regional connectome comparison

PBS vs cisplatin treatment	
Region	p-value
Thalamus L	0.01
Striatum L	0.02
Cortical Subplate L	0.04
Pallidum L	0.04
Vermis R	0.05

Cisplatin vs cisplatin + MSC treatment	
Region	p-value
Ectorhinal R	0.03

Next, we compared the connectome of cisplatin-treated mice with mice that had received MSC. Nasal administration of MSC increased the characteristic path length when compared to cisplatin-treatment alone (p = 0.04). Regionally, none of the above areas that were affected by cisplatin alone, were significantly different from PBS in cisplatin + MSC-treated mice. The right ectorhinal area showed higher clustering in cisplatin + MSC mice (p < 0.04) as compared to cisplatin-treated mice (Table 1).

### Nasally administered MSC restore white matter integrity

**Figure 4:**
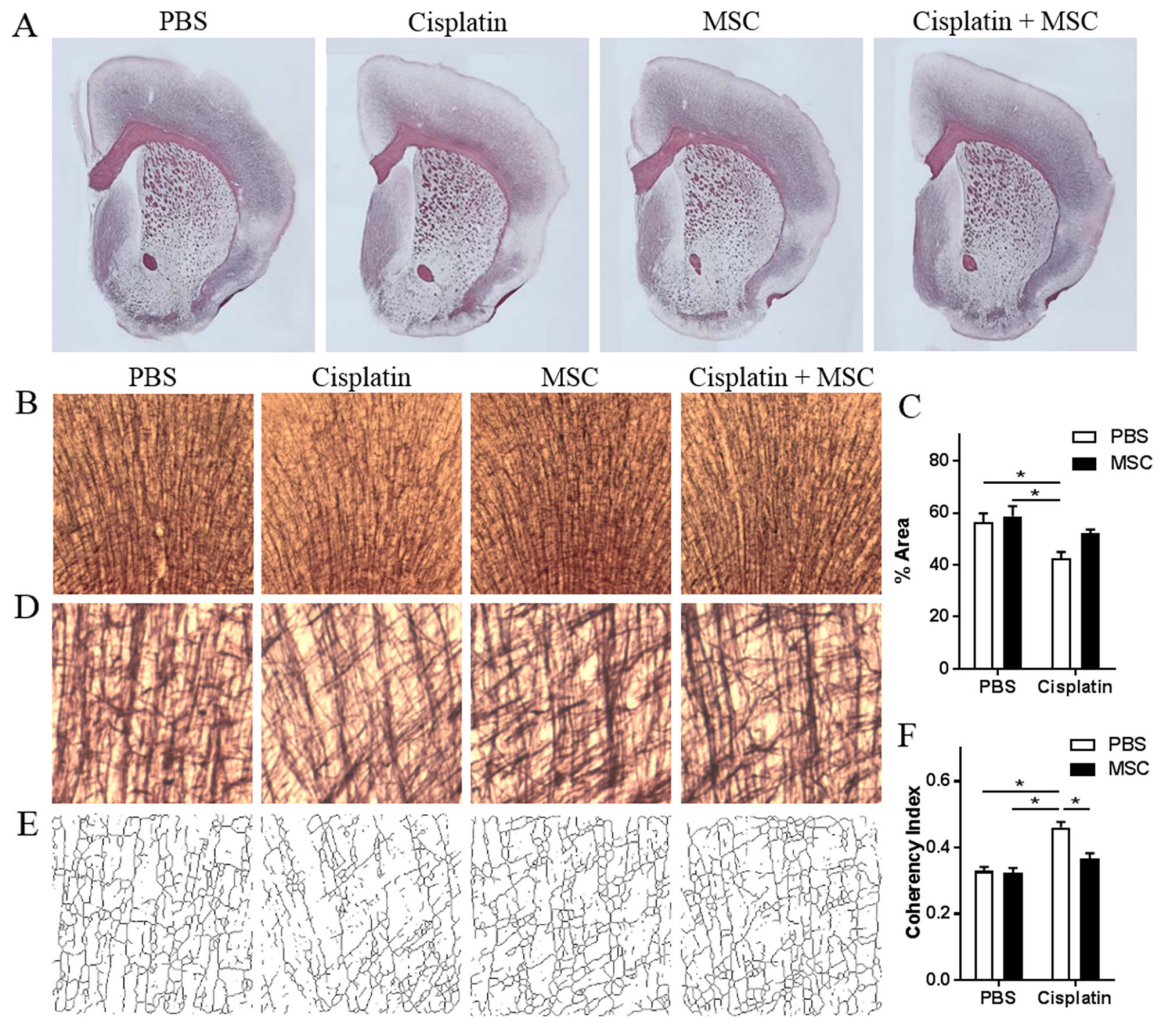
Effects of nasally administered MSC to cisplatin-treated mice on white matter integrity **(A)** Representative images of Black Gold II staining for myelin in brains of mice treated with PBS or cisplatin for two 5-day cycles followed by nasal administration of PBS or MSC as in Figure 1. **(B)** Black Gold II staining in cingulate cortex in animals treated with PBS, MSC, cisplatin, or cisplatin + MSC. **(C)** Percent area stained for Black Gold II was measured. **(D)** Higher magnification (20x) of myelin fibers in the cingulate cortex was **(E)** skeletonized, and the coherency index was measured **(F)** to assess the complexity of cortical myelination. Results are expressed as means ± SEM; n = 3 per group. Two-way ANOVA, *Tukey post hoc:* ^*^P < 0.05.

We next examined whether cisplatin induces morphological abnormalities in the white matter using Black Gold staining [29]. Histological assessment of the brain after cisplatin treatment showed a global disruption of myelination (Figure 4A). More specifically, there was a significant loss of myelin density in in the cingulate cortex (Figure 4B–4C). These deficits were normalized in response to nasal MSC administration (Figure 4A–4C). Cisplatin induced an increase in fiber coherency in the cingulate cortex, indicating reduced myelin arborization. MSC administration also normalized this decrease in coherency in the cingulate cortex (Figure 4D–4F).

### Effects of nasally administered MSC on the hippocampal transcriptome

**Figure 5:**
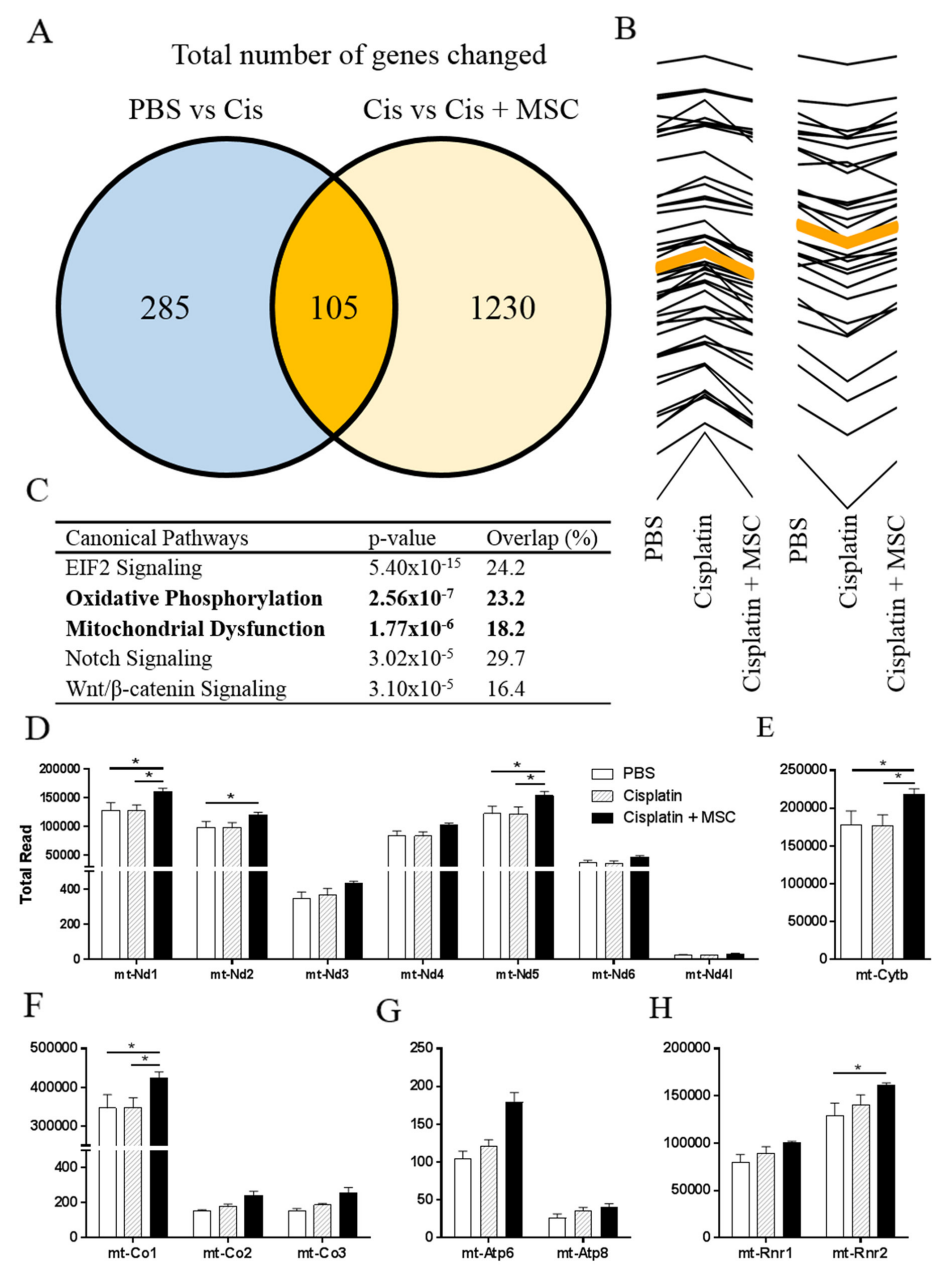
Effects of nasally administered MSC to cisplatin-treated mice on the hippocampal transcriptome Mice were treated with cisplatin for two 5-day cycles followed by nasal administration of MSC at 48- and 96-h post cisplatin. Total hippocampal RNA was collected at 72 h after the last dose of MSC. **(A)** Expression of a total of 390 genes was changed due to cisplatin treatment when compared to PBS controls. A total of 1335 genes were changed in response to nasal administration of MSC to cisplatin-treated mice when compared to mice treated with cisplatin alone. **(B)** Of the 105 shared genes that were changed due to cisplatin and cisplatin + MSC, 45 genes increased due to cisplatin treatment and were normalized after nasal MSC treatment. 30 genes decreased due to cisplatin treatment and were normalized after nasal MSC treatment. **(C)** The top 5 canonical pathways altered by administration of MSC to cisplatin treated mice (comparison of cisplatin alone versus cisplatin + MSC) as analyzed by Ingenuity Pathway Analysis. Bolded are the two pathways related to mitochondrial function. **(D–H)** Expression of mitochondrially encoded genes. The effect of nasal MSC administration to cisplatin-treated mice on total read numbers is depicted for **(D)** complex I, **(E)** complex III, **(F)** complex IV, **(G)** ATP Synthase, and **(H)** rRNA. Results are expressed as means ± SEM; n = 3. One-way ANOVA, *Tukey post hoc:* ^*^P < 0.05.

Transcriptional changes induced by cisplatin and MSC in the hippocampus at 7 days after the last dose of cisplatin (3 days after the last dose of MSC) were analyzed using RNA sequencing. Supplementary Figure 4A depicts a heat map of the differentially expressed genes (adjusted p<0.05). Cisplatin altered expression of 390 genes as compared to the saline control group. In addition, 1335 genes differed in expression when comparing the cisplatin + MSC group to the cisplatin alone group. 105 of the genes that were differentially expressed in response to cisplatin also changed in expression in response to administration of MSC to cisplatin-treated mice (Figure 5A). Out of these 105 overlapping genes, 45 were increased by cisplatin and reduced in response to MSC, while 30 were decreased by cisplatin and increased in response to MSC (Figure 5B and Supplementary Tables 1 and 2 for a list of these 75 genes). Since MSC were no longer detectable in the brain at 72 hrs after administration (as confirmed by the absence of the GFP transgene in the hippocampus at 72 hrs and 7 days after administration), we propose that these changes in gene expression reflect effects of the administered MSC on gene expression by the host.

We next performed pathway analysis to get more insight in the effect of nasally administered MSC on gene expression profiles in the brain of cisplatin treated mice. Interestingly, two of the top five canonical pathways identified (“Oxidative Phosphorylation” and “Mitochondrial dysfunction”) indicated changes in expression of genes involved in bioenergetic pathways (Figure 5C). Details on the specific genes that were differentially expressed in the 5 top pathways are presented in Supplementary Figure 5. A closer look at the two bioenergetic pathways shows that MSC administration to cisplatin-treated mice changed the expression of 29 nuclear encoded genes associated with mitochondrial function (27 upregulated and 2 downregulated). Next, we examined the effects of cisplatin and MSC treatment on the expression of the 13 mitochondrial encoded genes. Changes were detected in expression of mitochondrial genes encoding for complex I (Figure 5D), complex III (Figure 5E), and complex IV (Figure 5F), while expression of the mitochondrial genes encoding ATP synthases was not changed (Figure 5G). In addition, MSC treatment increased expression of one of the genes encoding mitochondrial ribosomal RNA (Figure 5H).

### Effects of nasally administered MSC on cisplatin-induced changes in mitochondrial respiratory function and morphology

**Figure 6:**
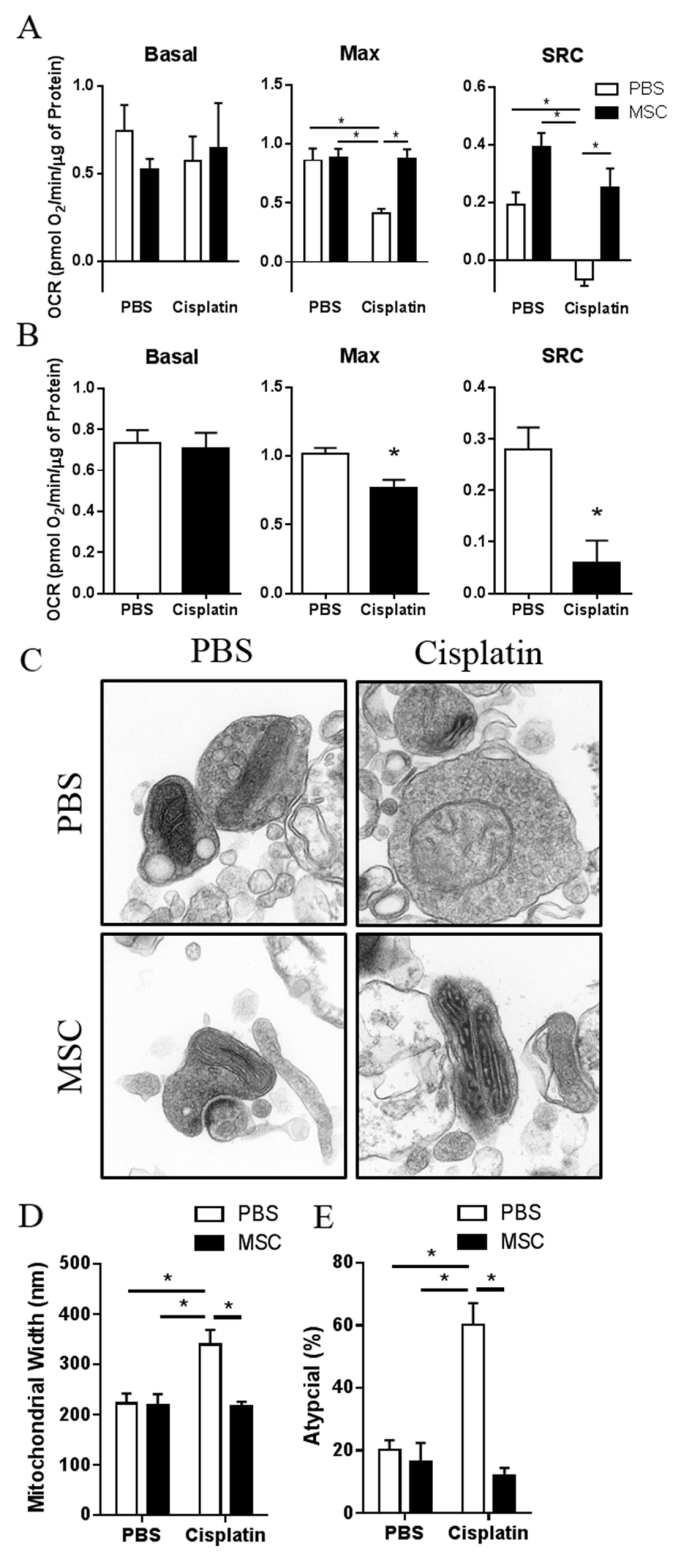
Effects of nasally administered MSC on cisplatin-induced changes in mitochondrial respiratory function and mitochondrial morphology **(A)** Mice were treated with cisplatin for two 5-day cycles followed by 2 doses of 1×10^6^ MSC nasally at 48 h and 96 h post cisplatin. Synaptosomes were isolated 3 weeks after the last dose of MSC. Results are expressed as means ± SEM; n = 10. Two-way ANOVA, *Tukey post hoc:* ^*^P < 0.05 versus PBS controls. Basal, basal respiration; Max, maximal respiratory capacity; SRC, Spare respiratory capacity. **(B)** At 48 h after completion of cisplatin treatment, synaptosomes were isolated from the brains of animals treated with cisplatin for two 5-day cycles. Oxygen consumption rates were analyzed using the Seahorse XFe 24 Analyzer. Basal, max, and SRC were calculated. Results are expressed as means ± SEM; n = 5. *T*-test: ^*^P < 0.05. Basal: p = 0.6394; Max: p = 0.0116; SRC: p = 0.0071. **(C)** Synaptosomes were isolated from the brains of mice treated with PBS, cisplatin, MSC, or cisplatin + MSC at 3 weeks after the last dose of MSC. **(D)** Mitochondrial width and **(E)** and percentage of atypical mitochondria were quantified. Results are expressed as means ± SEM; n = 4. Two-way ANOVA, *Tukey post hoc:* ^*^P < 0.05 versus PBS controls.

Our RNA sequencing analysis identified changes in the expression of genes involved in mitochondrial functions as top pathways that had changed after administration of MSC to cisplatin-treated mice. In addition, we demonstrated recently that cisplatin-induced cognitive deficits are associated with structural and functional mitochondrial abnormalities in brain synaptosomes [15]. Seahorse analysis of synaptosomes demonstrates that cisplatin-treated mice showed reduced maximal and spare respiratory capacity 3 weeks after the last dose of MSC. Administration of MSC led to a full recovery of the cisplatin-induced impairment in maximal and spare respiratory capacity (Figure 6A).

To determine whether nasal MSC treatment reverses already existing mitochondrial deficiencies or prevents mitochondrial damage, we also tested mitochondrial respiratory function at 48 h after completion of cisplatin treatment (Figure 6B), which is the time point before MSC administration. At 48 h after cisplatin treatment, we already detected a marked decrease in maximal respiration and spare respiratory capacity in the synaptosomes from cisplatin-treated mice, suggesting that nasal MSC treatment truly reverses existing mitochondrial deficiencies.

The results in Figure 6 show that nasal MSC administration also restored mitochondrial morphology (Figure 6C–6E); the cisplatin-induced increase in mitochondrial width (Figure 6D) as well as the increase in the percentage of atypical mitochondria (Figure 6E) were reversed by nasal MSC administration to cisplatin-treated mice.

### Nasally administered MSC after completion of cisplatin treatment did not promote tumor growth

**Figure 7:**
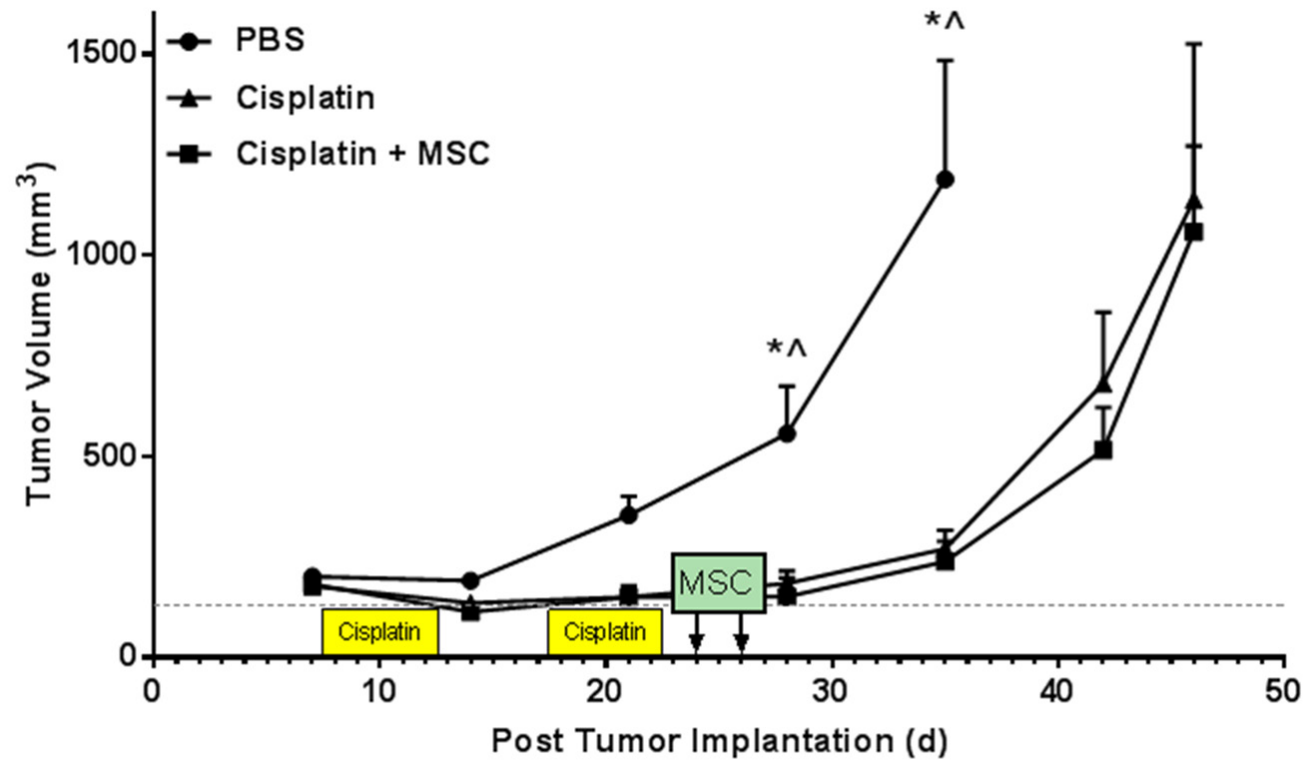
Effects of cisplatin and nasally administered MSC on tumor growth Mice were implanted with mEER tumors in the hind leg. 7 days post-implantation animals were treated with cisplatin or PBS for two 5-day cycles followed by nasal administration of PBS or MSC as in all other experiments. Tumor volumes were measured during and after cisplatin and MSC treatment. Results are expressed as means ± SEM; n = 5. Two-way ANOVA, *Tukey post hoc:* ^*^P < 0.05 versus cisplatin. ^^^*P* < 0.05 versus cisplatin + MSC.

While unlikely, it is possible that MSC or factors released by MSC could be released into the periphery and interfere with the anti-tumor effects of cisplatin and/or promote tumor growth. Therefore, to assess the potential interference of nasal administration of MSC with cancer treatment, we used a heterotopic syngeneic HPV-related head and neck tumor model. mEER cells (5 x 10^4^ cells) were injected into the hind leg. Seven days after tumor cell implantation, mice were treated with cisplatin or PBS followed by MSC treatment using the same schedule as above. Cisplatin treatment significantly delayed tumor growth as compared to controls. Nasal administration of MSC after completion of cisplatin treatment did not affect tumor growth (Figure 7).

## DISCUSSION

In the present study, we show for the first time that nasal administration of MSC promotes recovery from cisplatin-induced cognitive impairments including deficits in working memory, spatial recognition, and executive functioning in both male and female mice. Furthermore, we show for the first time that cisplatin administration induces a decrease in global functional neuronal connectivity in the mouse brain as measured by rsfMRI. Nasal MSC administration reverses the abnormal characteristic path length observed as a result of cisplatin treatment. RNA sequencing analysis of the hippocampus of cisplatin-treated mice revealed that MSC administration increased expression of nuclear and mitochondrially encoded genes involved in mitochondrial respiration. Consistently, nasally administered MSC repaired existing synaptosomal mitochondrial dysfunction and abnormal mitochondrial morphology in cisplatin-treated mice, indicating a true restoration of function by MSC. The cisplatin-induced structural changes in cortical myelination were also completely restored by nasal MSC administration. Finally, we show that nasal MSC administration does not have any effect on tumor growth in cisplatin-treated mice.

Nasal administration of MSC is a non-invasive strategy that could be of great benefit for clinical practice [30]. Apart from the easy route of administration, it is superior to intravenous (i.v.) injections, which require many more MSC and have the disadvantage that MSC will end up for the greater part in peripheral organs like lung and liver [31]. Indeed, preliminary evidence we obtained in a model of cognitive impairment induced by cranial irradiation and temozolamide indicates that nasal administration of MSC improves cognitive function, while intravenous administration of MSC, even when given at twice the dose, is not effective (unpublished data, Chiu et al.)

Nasally administered MSC migrated into the brains of the cisplatin-treated mice where fluorescinating MSC could be found in several brain areas, including the hippocampus, thalamus, and cortex. We did not detect MSC in the brains of PBS-treated mice (data not shown) which may imply that MSC are specifically signaled to migrate into brain areas where there is cisplatin-induced damage. Indeed, cisplatin causes abnormalities in myelin staining in cortical areas (Figure 4 and [14, 15]) as well as damage to proliferating neuronal precursors in hippocampus and the subventricular zone [15]. In this respect, it is of interest that after unilateral neonatal hypoxia-ischemia, nasally administered MSC also migrate into the brain but exclusively to lesioned hemisphere even when administered to both nostrils [18, 32].

Unbiased RNA sequencing analysis revealed that administration of MSC increased the expression of nuclear encoded genes for components of the four complexes of the electron transport chain. When analyzing mitochondrial RNA, a similar pattern emerged, showing that expression of genes encoding components of complex I, III, and IV of the electron transport chain were upregulated. Functionally, MSC administration normalized the cisplatin-induced mitochondrial damage as measured by oxygen consumption rate and also normalized the mitochondrial morphology. We previously reported that neuronal mitochondrial dysfunction is the mechanism underlying cisplatin-induced cognitive dysfunction and the associated brain damage [15]. Specifically, we showed that the spare respiratory capacity of mitochondria in synaptosomes of cisplatin-treated mice is diminished. However, co-administration of the mitochondrial protectant pifithrin-μ, which prevents the translocation of p53 to the mitochondria, prevented mitochondrial abnormalities, the cisplatin-induced cognitive dysfunction and the associated brain damage [15]. The latter data suggest that mitochondrial dysfunction is the underlying mechanism of cognitive impairment as a result of cisplatin.

In line with its mechanism of action, pifithrin-μ did not reverse existing cognitive impairment but only prevented the cognitive dysfunction. Here, we show that nasal MSC treatment has the advantage of being capable of reversing already existing damage as the mitochondrial dysfunction had already fully developed at the time of MSC administration 48 h after completion of the full regimen of cisplatin injections. Restoration of synaptosomal mitochondrial function to control levels in response to nasal MSC administration was associated with complete recovery of cognitive abilities. Together, these findings suggest that MSC act by balancing the bioenergetic machinery allowing repair of cognitive functions.

Current research in cancer survivors suggests that the use of chemotherapeutics is associated with decreased functional and structural connectivity in the brain [7–9]. In line with these findings, we here present the first evidence that in mice cisplatin treatment affects functional network connectivity as measured with rsfMRI. Characteristic path length is a measure of the connection between various regions of the brain, where decreases in path length are associated with lower overall cognitive ability [5–7]. Our data suggest that the brain network is overly integrated in cisplatin-treated mice and more closely resembles that of random, noisy networks, which is consistent with the higher density of connectomes in the cisplatin group. Nasal MSC administration increased the path length in cisplatin-treated mice, indicating normalization of the functional connectome which underlines the powerful action of nasal MSC administration. Only the right ectorhinal area showed higher clustering in cisplatin + MSC mice (p = 0.03) as compared to cisplatin-only mice. This hyper-connectivity may reflect some connectome remodeling associated with MSC and/or some over-correction of cisplatin-related injury. The structural changes in myelination including fiber complexity and global density of myelin as a result of cisplatin were also completely restored by MSC indicating that the regenerative capacities of MSC are powerful and may involve repair of a shared mechanism of cisplatin-induced brain damage.

We showed before that MSC administered either via the nose or directly via cranial administration as a treatment for neonatal hypoxic-ischemic brain damage do not integrate into the brain but merely act by boosting endogenous repair mechanisms leading to lesion repair and restoration of cognitive and motoric function [17, 19, 24]. MSC genetically overexpressing green fluorescent protein could only be found 48-72 h after administration either by immune fluorescence or by genomic DNA analysis. When investigating migration of MSC into cisplatin-treated brains, we could only detect MSC until 24 h after administration (data not shown). Consistently, MSC administered after cisplatin are no longer detectable at 72 hrs after the last dose; MSC isolated from GFP+ animals were detected in the brain by 12-hrs but were undetectable by 72-hrs, suggesting that the long-lasting effects of MSC is due to a promotion of endogenous recovery, and most likely the MSC did not transdifferentiate into another cell type such as neurons. It has been reported that MSC injected intravenously for other clinical purposes such as treatment to reduce the infarct size after a myocardial infarction, have a half-life of only 24 h as well [33]. We therefore propose that MSC do not survive long and do not integrate into the host in the cisplatin-treated brain, which is important from a perspective of safety. Importantly, MSC administration after cisplatin treatment did not affect tumor growth in cisplatin-treated mice, indicating that it is a safe intervention.

Taken together, our results indicate that nasal administration of MSC is an effective treatment for chemobrain as a result of cisplatin treatment. Nasal administration of MSC restores executive function and memory after cisplatin treatment. This reversal of cognitive deficits is associated with a restoration of cortical myelination and functional connectivity between brain regions. MSC act via repairing synaptic mitochondrial function as shown by a repair of the morphology and oxygen consumption rates of mitochondria. Therefore, nasal MSC administration may be considered as an important therapeutic strategy to treat established cancer-treatment-induced cognitive impairment.

## MATERIALS AND METHODS

### Animals

C57BL/6J male and female mice (Jackson Laboratory) aged 9 weeks were housed at 22 ± 2°C, on a 12/12 h reverse dark–light cycle (dark 830–2030 h) with water and food *ad libitum*. Video recording of animal behavior was performed under red light using a Bell & Howell Rogue Night Vision Digital Video Camcorder. C57BL/6-Tg (UBC-GFP) 30Scha/J male mice (The Jackson Laboratory) were used as a source for bone marrow-derived GFP^+^ MSC. All experiments were conducted at MD Anderson Cancer Center in Houston, Texas in accordance with Institutional Animal Care and Use Committee-approved protocols.

### Experimental design

Mice were randomly assigned to experimental groups and behavioral tests were performed by an independent investigator blinded to treatments. Videos were scored by investigators blinded to the experimental set up. For all power analyses, we did set the type I error at 0.05 and the power at 80%. The minimal change to be detected was set at 25%.

### Chemotherapy and MSC treatment

Cisplatin (2.3 mg/kg/day; Fresenius Kabi USA, Lake Zurich, IL) or phosphate-buffered saline (PBS) was administered intraperitoneally daily for 5 days, followed by a 5-day rest without injections and another 5-day injection cycle [15]. This dose regimen of cisplatin is similar in the cumulative dose used in human cancer patients. Mouse MSC were purchased from Invitrogen (GIBCO Mouse C57BL/6, Invitrogen, Carlsbad, CA) or isolated from bone marrow of C57BL/6-Tg (UBC-GFP) 30Scha/J animals as previously described [18]. MSC were cultured in Dulbecco’s Modified Eagle’s medium/F12 medium with GlutaMax-I, supplemented with 10% MSC-qualified fetal Bovine Serum (FBS) and 5 μg/mL gentamycin (GIBCO Life Technology, Invitrogen, Carlsbad, CA).

Thirty minutes before administration of MSC, 3 μl per nostril of hyaluronidase in PBS (total 100 U per mouse, Sigma-Aldrich, St. Louis, MO) was administered to each nostril to increase the permeability of the nasal mucosa [23, 24]. Mice received MSC in a volume of 3 μL, twice applied to each nostril with a total volume of 12 μL or PBS. Based on previous studies in neonatal brain damage, administration of MSC around 3 days after injury is optimal for recovery [17, 23, 24, 27, 32]. We first determined the optimal dose of MSC in a range of 0.125-1×10^6^ MSC/ mouse/day administered 48 and 96 h after cisplatin treatment. Robust effects were observed at a dose of 1×10^6^ MSC/mouse/day in the PBT (not shown). This dose was therefore selected for further study.

For cell tracking studies, MSC were labeled with PKH-26 Red fluorescent cell linker kit (Sigma-Aldrich, St. Louis, MO) or with CTGB (green) (Life technologies, Carlsbad, CA) following manufacturer’s instructions. MSC from GFP-transgenic mice were used for assessment of survival of MSC and/or progenitors in the brain by real time PCR analysis of the presence of the transgene.

### Behavioral testing

To assess cognitive function, we tested mice using the puzzle box test (PBT), the novel object/place recognition test (NOPRT), and Y-maze test 7-10 days after MSC administration.

### Puzzle box test

The PBT, which consists of 11 trials at three levels of complexity, is used as a measure of executive functioning [28]. Cisplatin-treated and control mice were submitted to the PBT 7–10 days after the last MSC/PBS administration. Mice were transferred to a testing arena (71 cm x 28 cm) containing a lighted (55 cm x 28 cm) and a dark compartment (15 cm x 28 cm) separated by a wall with a connecting tunnel (4 cm x 2.5 cm). Mice were introduced to the lighted side, and in a series of trials with increasing difficulty, the time to enter the preferred dark compartment was recorded. During the “easy” trials the tunnel was freely accessible. During the “intermediate” trials, the tunnel was filled with bedding, requiring the mice to burrow through to enter the dark compartment. During the “difficult” trials, the tunnel was closed with a cardboard lid that the mice were required to remove before they could enter the tunnel. Testing occurred over 4 days; three consecutive easy trials on day one (trials 1-3), one easy trial (trial 4), followed by two intermediate trials on day 2 (trials 5 and 6), one intermediate trial (trial 7), followed by two difficult trials (trials 8, 9) on day 3, and two additional difficult trials (trial 10, 11) on day 4 [28].

### Novel object/place recognition test

This test was performed as previously described [14, 15]. In brief, mice were introduced to two identical objects for 5 min (training phase) and then returned to their home cages. After 30 min. mice were returned to the arena which contained one now-familiar object in the same location as in the training session, and one novel object placed on the opposite end of the arena. Investigative behavior toward either object during the 5-min testing period was evaluated using EthoVision XT 10.1 video tracking software (Noldus Information Technology Inc., Leesburg, VA). Discrimination index was determined by the equation

$$\left({\text{T}}_{\text{Novel}}-{\text{T}}_{\text{Familiar}}\right)\text{/}\left({\text{T}}_{\text{Novel}}+{\text{T}}_{\text{Familiar}}\right).$$

### Y-Maze test

This test was performed as previously described [15, 34]. Mice were randomly placed in one of the arms of a symmetrical three-arm, gray plastic Y-maze with external spatial room cues. Movement was recorded for 5 min., and the percentage of perfect alternations defined as exploration of all three arms sequentially before reentering a previously visited arm was calculated.

### Resting-state fMRI

To test whether cisplatin induces changes in the functional connectome, mice underwent rsfMRI using a 7 Tesla Bruker BioSpec small animal scanner while mice were anesthetized using isoflurane. Isoflurane was administered at 1% (mixed with O_2_) to keep the respiration rate between 80 and 120 beats per minute [35]. Mice were secured into the head coil with a bite bar and the head was taped down to minimize motion. We first acquired a single-shot gradient, axial echo planar imaging (EPI) functional sequence [slice thickness = 0.5 mm, gap = 0.0 mm, repetition time (TR) = 2000 ms, echo time (TE) = 12 ms, matrix = 80×64×32, field of view (FOV) = 20×16 mm, flip angle = 75°, number of volumes = 450, averages = 1, scan time = 15 min] followed by a T2-weighted, turbo spin echo, rapid acquisition with refocused echoes (Turbo RARE) sequence (slice thickness: 0.5 mm, gap = 0.0 mm, TR = 4000 ms, TE = 40.00 ms, matrix = 256×180, FOV = 26.600×18.000 mm, flip angle = 90°, number of images = 32, scan time = 4 mins. and 24 secs).

RsfMRI volumes were preprocessed to reduce noise (motion, signal and physiological artifacts) [36] and restrict the signal to the low frequency range (< 0.1 Hz) associated with intrinsic functional networks [37]. Preprocessing included spatial normalization of rsfMRI volumes via a co-registered T2-weighted volume to the Allen Mouse Brain atlas [38]. Using regions from this template, we computed a 62×62 correlation matrix for each mouse.

We then applied graph theoretical analysis using the Brain Connectivity Toolbox [39] and in-house software (https://github.com/srkesler/bnets) to measure connectome properties. Connectomes are graphs that model the brain network as a system of nodes (regions) and edges (connections) [40]. Connectomics provides unique insight regarding the brain’s topological organization [40]. We measured the normalized clustering coefficient, characteristic path length, small-worldness index, global/local efficiency, and density of each mouse connectome [39, 40].

Functional connectomes were constructed for each subject with N = 62 nodes, network degree of *E* = number of edges and a network density of *D* = *E*/[(*N* × (*N*-1))/2] representing the fraction of present connections to all possible connections. Negative functional edges were zeroed given evidence that properties of negative correlation networks are different than those of positive correlation networks [41]. We evaluated weighted networks without any thresholding. Because individual variation in network density can affect connectome properties [42], connectome properties were compared between groups using the general linear model with network density as a covariate [43]. We also examined regional effects by measuring and comparing clustering coefficient of all nodes in the networks at minimum connection density using Wilcoxon ranksum test. Data from one mouse in the cisplatin-MSC group and one in the PBS-MSC group had to be excluded from analyses due to lack of small-worldness organization (i.e. small-worldness index was less than one) [44]. This may have been due to the effects of anesthesia which can affect individual mice differently and is known to affect functional connectivity, although we did not observe it in the control mice [45].

### RNA sequencing

Transcriptional changes induced by cisplatin and MSC in the hippocampus were investigated using whole-genome RNA sequencing. RNA was collected 72 h after the last dose of MSC. Total RNA was isolated using the RNeasy MinElute Cleanup Kit (Qiagen, Hilden, Germany) and analyzed by the RNA Sequencing Core Lab personnel at MD Anderson Cancer Center. The sample libraries were generated using the Stranded mRNA-Seq kit (Kapa Biosystems, Wilmington, MA) following the manufacturer’s guidelines. One lane in a 75-nt paired-end run format was performed using a HiSeq 4000 Sequencer.

Data analysis was performed using a comprehensive in-house RNASeq pipeline. We used STAR to align paired-end reads to the mm10 version of the mouse reference genome, feature count to obtain expression counts of genes and eons, and cufflink to estimate gene expression. Genetic variants and fusion candidates were detected and annotated using GATK unified genotypes, snpEff, STAR and Oncofuse but were not explored in this study. Qualify of raw and aligned reads was assessed using FastQC and qualimap. Expression data of 9 samples were analyzed in R using bioconductor packages such as DESeq2, biomart. For geneses enrichment analyses, we separated differentially expressed genes between PBS and Cisplatin groups into up and down regulated genes and compared them with the log2 fold change of expressions between Cisplatin and Cisplatin + MSC groups, using 1000 permutations on phenotype.

We analyzed the genes that were differentially expressed (adjusted p<0.05) in the hippocampus of cisplatin treated mice who did and did not receive MSC using Ingenuity Pathway Analysis (IPA; Qiagen Inc., https://www.qiagenbioinformatics.com/products/ingenuity-pathway-analysis/). Expression of mitochondrial encoded genes is presented as average total mitochondrial RNA read ± SEM and analyzed using a One-Way ANOVA.

### Tissue processing and Black Gold II staining

Brains were fixed in 4% PFA and frozen sections were taken at 25 μm. Myelin staining was performed using Black Gold II (AG105, Millipore) according to manufacturer’s instructions [29]. Free floating sections were mounted onto slides and dried overnight at room temperature. Slides were rehydrated in miliQ water and immersed in Black Gold II at 60°C for 16 minutes. Following washes in water, slides were transferred to prewarmed 1% sodium thiosulfate for 3 minutes at 60°C. Slides were then rinsed and dehydrated through a series of ethanol and xylene and cover slipped with Permount. Bright field. Images of myelin were quantified using ImageJ.

### Mitochondrial function and morphology

Mitochondrial function and morphology were analyzed in synaptosomes isolated from total brain [15]. Synaptosomes were resuspended in base media (Seahorse Biosciences/Agilent Technologies, Santa Clara, CA) supplemented with 11 mM glucose, 2 mM glutamine, and 1 mM pyruvate. 75 μg of total protein was plated in a Seahorse XFe 24 microplate (Seahorse Biosciences) pre-coated with GelTrex (1 h in 37°C; Life Technologies/Thermo Fisher Scientific, Waltham, MA). Oxygen consumption rate at baseline and the response to 4 μM oligomycin, 6 μM carbonyl cyanide 4-(trifluoromethoxy)phenylhydrazone (FCCP), and 2 μM of rotenone and 2 μM of antimycin A were determined. All values were corrected for non-mitochondrial oxygen consumption.

For TEM analysis of mitochondrial morphology, synaptosomes were fixed in 2% glutaraldehyde plus 2% PFA in PBS and processed [15, 46]. Quantification of mitochondrial morphology was performed by two independent researchers blinded to treatment using ImageJ. Mitochondrial width was defined as the measurement perpendicular to the orientation of the cristae. Atypical mitochondria were defined as those with a mitochondrial width greater than 300 nm and opacity less than 50%.

### MSC migration into brain

MSC labelled with PKH-26 (red) or CTGB (green), were administered 48 h after the last dose of cisplatin/PBS. Brains were harvested 12 h later. Frozen brain sections were examined by confocal microscopy for presence of labelled MSC. In a separate experiment, MSC were isolated from GFP transgenic mice and administered to cisplatin treated animals as before. The presence of GFP transgenic transcript in the brain was analyzed using qPCR with the forward Primer (AGT GCT TCA GCC GCT ACC), reverse primer (GAA GAT GGT GCG CTC CTG), and transgenic probe (TTC AAG TCC GCC ATG CCC GAA).

### Effect of nasal MSC administration after completion of cisplatin treatment on tumor growth

Mice were injected with a heterotopic syngeneic murine model of human papilloma virus (HPV)-related head and neck cancer into the hind leg (5 × 10^4^ cells) as we described before [9]. Tumor cells are derived from C57BL/6 oropharyngeal epithelial cells transfected with oncogenes E6/7 of HPV 16 and hRAS (mEER) [47, 48]. Seven days after tumor cell implantation, animals were treated with cisplatin or PBS and MSC using the same schedule as for the behavioral experiments (2.3 mg/kg) for two 5-day cycles followed by nasal MSC administration at 48 and 96 hours after completion of cisplatin treatment. Tumor volume was measured using a Vernier calipers from three mutually orthogonal tumor diameters, where volume = (π/6)(d1^*^d2^*^d3) [47, 48].

### Statistical analysis of behavioral, white matter staining and mitochondrial data

Data are presented as mean ± standard error of the mean (SEM). Data were analyzed using GraphPad Prism 6 (GraphPad). One-way or two-way analysis of variance (ANOVA) was used with or without repeated measure to test for statistical significance where applicable, with alpha = 0.05. *Post hoc* pair-wise, multiple-comparisons were performed using the two-tailed Tukey’s test. Differences were considered statistically significant at *P* < 0.05.

## SUPPLEMENTARY MATERIALS FIGURES AND TABLES



## Abbreviations

CTGB: cell tracker green BODIPY
MSC: mesenchymal stem cells
NOPRT: novel object/place recognition test
rsfMRI: resting-state functional MRI
PBS: phosphate-buffered saline
PBT: puzzle box test
SEM: standard error of the mean
