# Supplementary material for: Nasal administration of mesenchymal stem cells restores cisplatin-induced cognitive impairment and brain damage in mice
Source: Oncotarget. 2018 Oct 30;9(85):35581–97. doi: 10.18632/oncotarget.26272 (PMC6238972; doi:10.18632/oncotarget.26272)
Supplement: Supplementary file 1 [file oncotarget-09-35581-s001.pdf]

Nasal administration of mesenchymal stem cells restores cisplatin-induced cognitive impairment and brain damage in mice

SUPPLEMENTARY MATERIALS

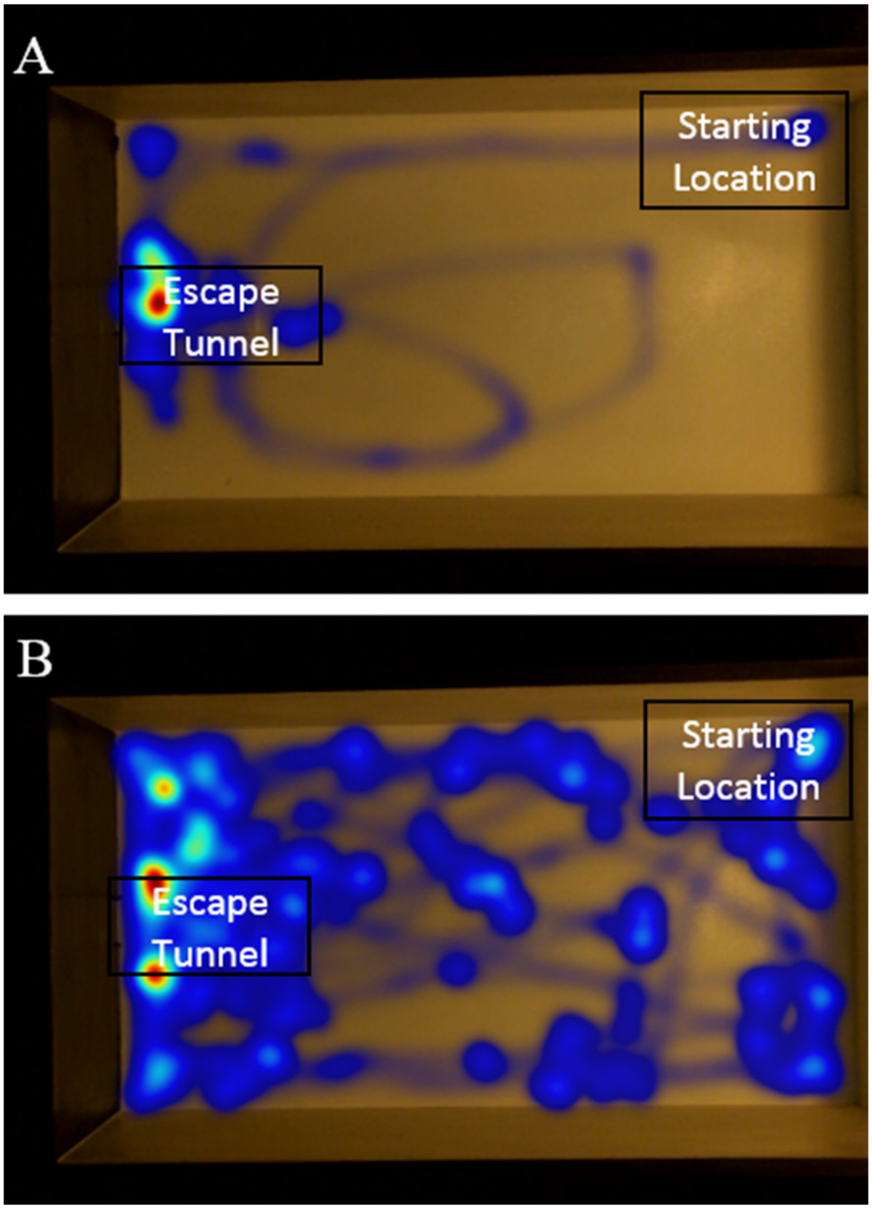

Supplementary Figure 1: Representative heat map of time spent in the light compartment of the puzzle box. Male mice were treated with cisplatin and MSC, and the PBT was performed as in Figure 1A. Representative heat map of time spent in the light compartment of the puzzle box of PBS **(A)** and cisplatin–treated **(B)** male mice in trial 8 (first difficult trial).

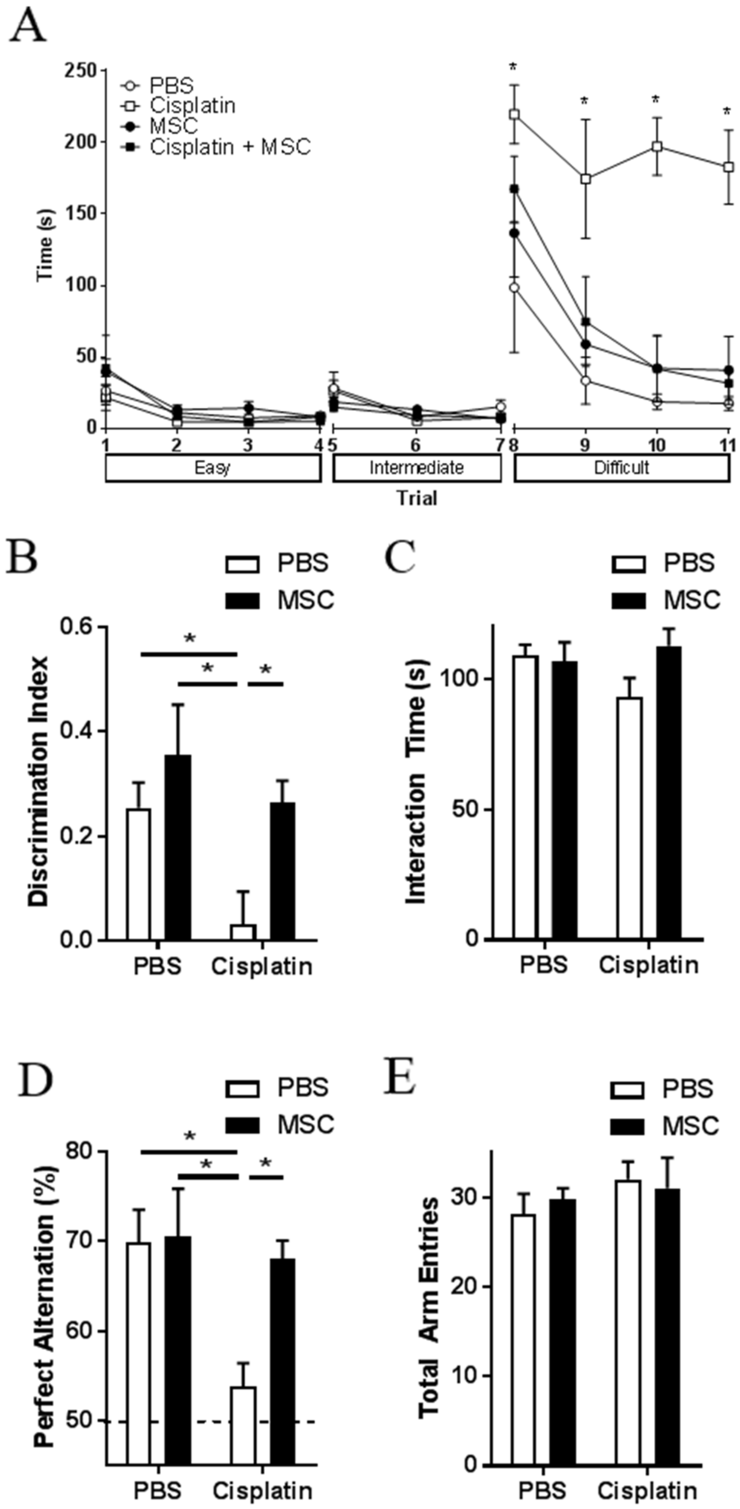

Supplementary Figure 2: Effect of nasally administered MSC on cisplatin-induced cognitive impairments in female mice. (A) Female mice were treated with cisplatin and MSC, and the PBT was performed as with the male mice in Figure 1A. n = 4-7. *Tukey post hoc:* $^{*}P < 0.05$ versus PBS controls. **(B)** Female mice were treated with cisplatin and MSC, and the NOPRT was performed. n = 4-7. *Tukey test:* $^{*}P < 0.05$ versus PBS controls. **(C)** Total interaction times in the NOPR test were not affected by cisplatin and MSC treatment in female mice. n = 4-7. Two-way ANOVA Cisplatin x MSC interaction. **(D)** The percentage spontaneous alternation in a Y-maze was determined 1 day after the completion of the NOPRT in female mice. Dotted line indicates random chance. Results are expressed as means ± SEM; n = 4-7. *Tukey test:* $^{*}P < 0.05$. **(E)** Total arm entries in the Y-maze were not affected by cisplatin and MSC treatment in female mice. n = 4-7.

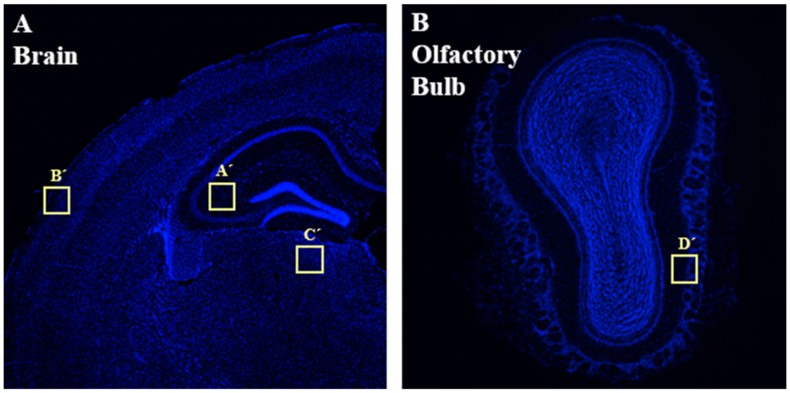

Supplementary Figure 3: Overview of location where MSC were detected. Overview of brain **(A)** and olfactory bulb **(B)** depicting the location of the images depicted in Figure 2 in the hippocampus (A'), cortex (B'), thalamus (C'), and olfactory bulb (D').

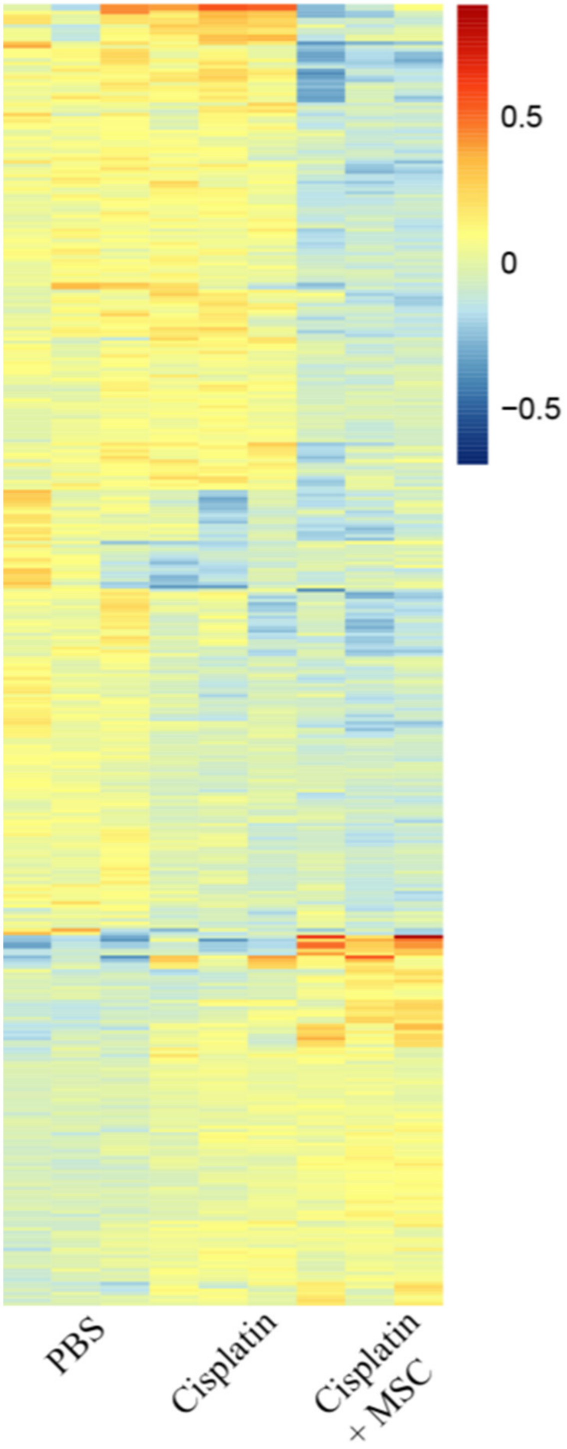

Supplementary Figure 4: Effects of cisplatin and MSC on gene expression. Heat map of differentially expressed genes between samples of PBS, cisplatin, and cisplatin + MSC treated animals (adjusted $p<0.05$).

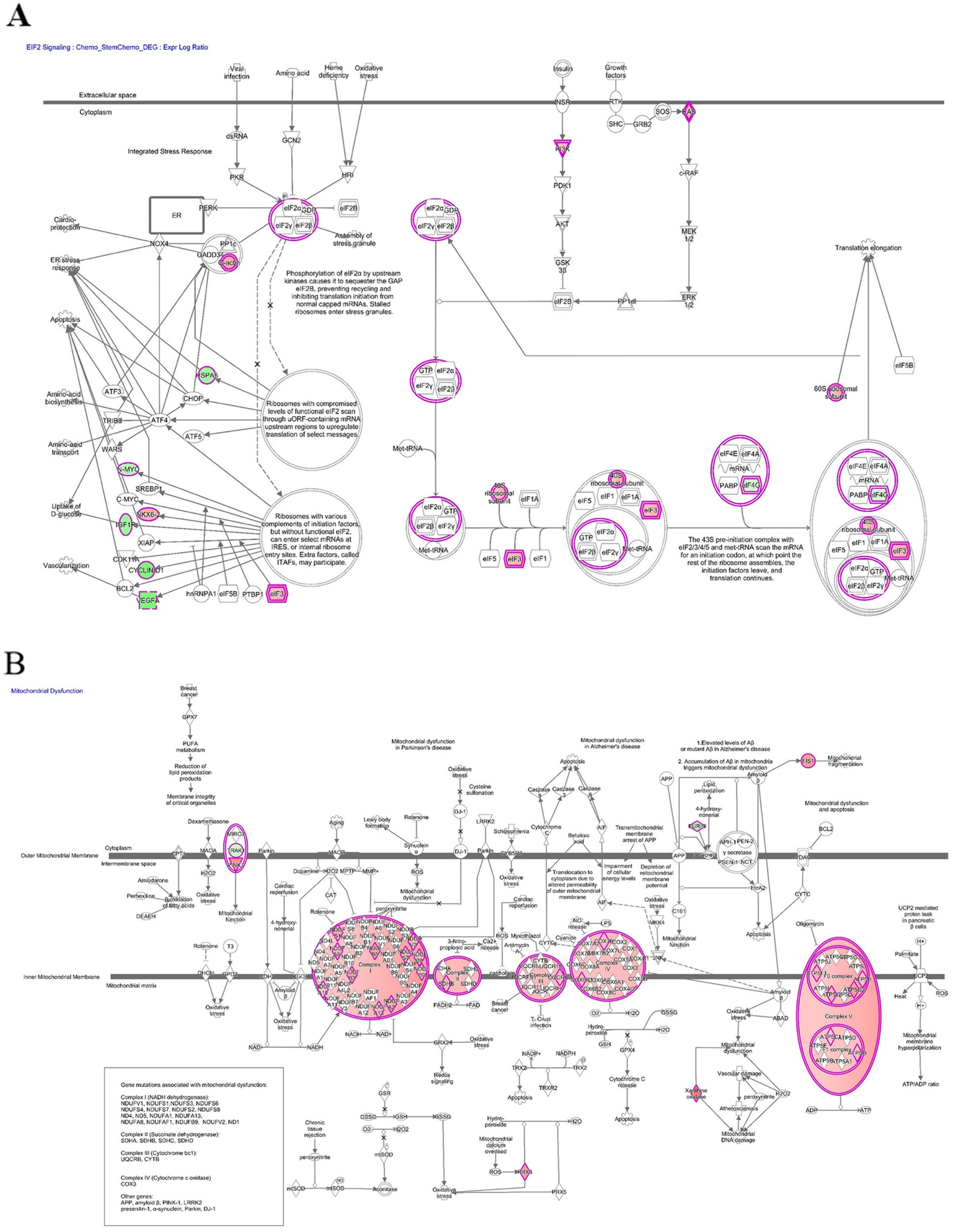

Supplementary Figure 5: Details on differentially expressed genes in the top five canonical pathways. Genes that were upregulated (red) or downregulated (green) in response to nasal MSC administration to cisplatin-treated mice for EIF2 signaling **(A)**, mitochondrial dysfunction and oxidative phosphorylation **(B)**

C

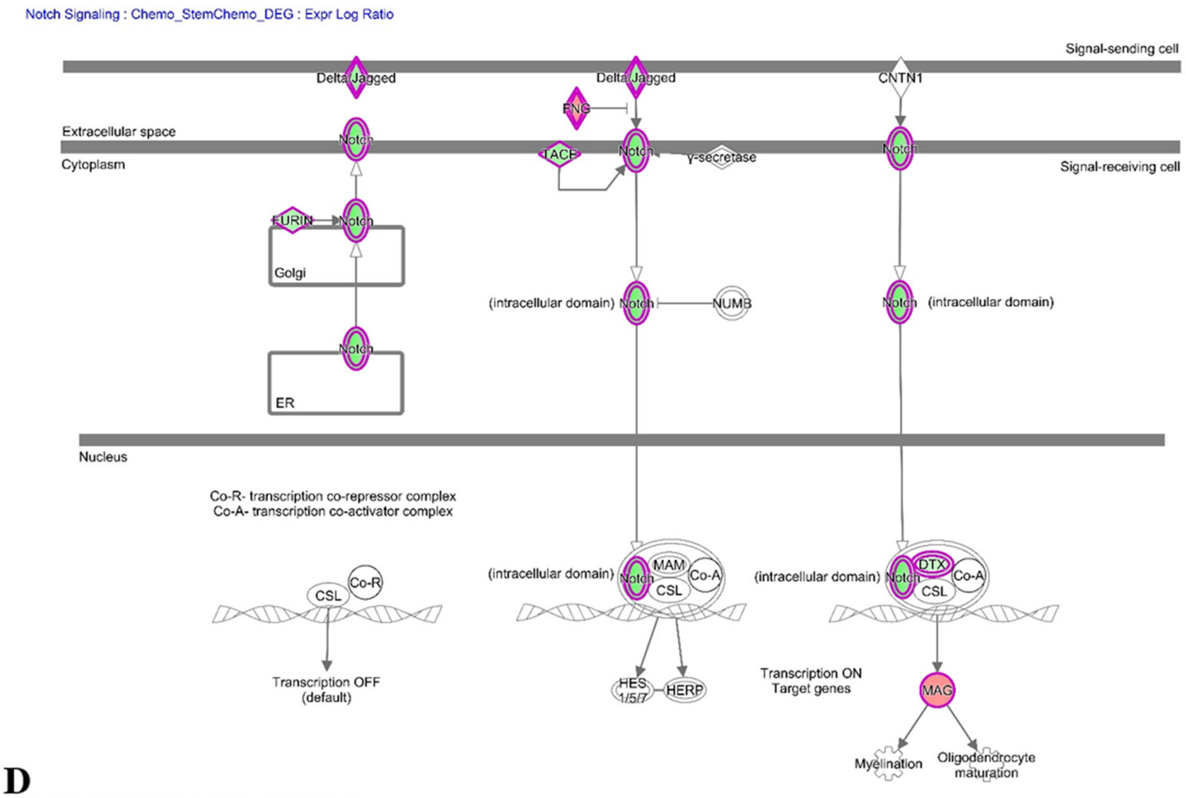

D

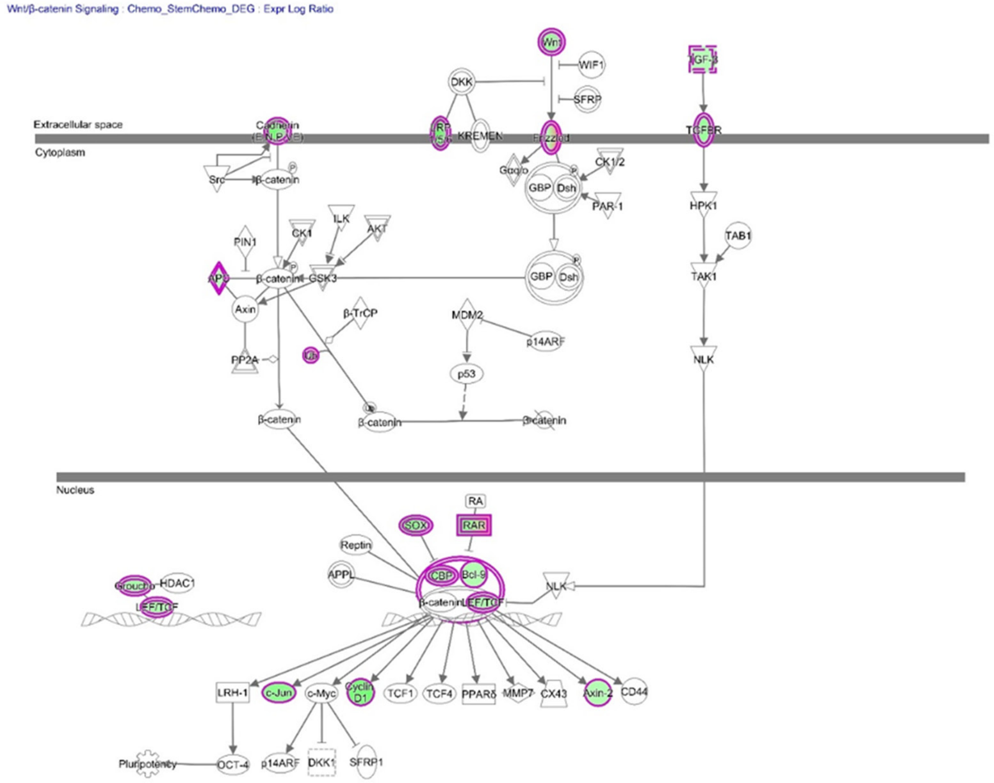

Supplementary Figure 5: (Continued) Details on differentially expressed genes in the top five canonical pathways. Notch signaling **(C)**, and Wnt/β-catenin signaling **(D)**.

Supplementary Table 1: Genes upregulated by cisplatin and normalized by nasal administration of MSCs

Symbol	Entrez Gene Name
ACKR1	atypical chemokine receptor 1 (Duffy blood group)
ARC	activity regulated cytoskeleton associated protein
AXIN2	axin 2
Brd4	bromodomain containing 4
BTG2	BTG anti-proliferation factor 2
CALR	calreticulin
CEACAM1	carcinoembryonic antigen related cell adhesion molecule 1
CMIP	c-Maf inducing protein
COL1A1	collagen type I alpha 1 chain
CTSC	cathepsin C
CYR61	cysteine rich angiogenic inducer 61
DUSP1	dual specificity phosphatase 1
DUSP18	dual specificity phosphatase 18
FGF10	fibroblast growth factor 10
FLT1	fms related tyrosine kinase 1
FOS	Fos proto-oncogene, AP-1 transcription factor subunit
GPR151	G protein-coupled receptor 151
HEXIM1	hexamethylene bisacetamide inducible 1
HSP90B1	heat shock protein 90 beta family member 1
HSPA5	heat shock protein family A (Hsp70) member 5
HSPB1	heat shock protein family B (small) member 1
HSPG2	heparan sulfate proteoglycan 2
IER2	immediate early response 2
IGF1R	insulin like growth factor 1 receptor
INHBB	inhibin beta B subunit
JUN	Jun proto-oncogene, AP-1 transcription factor subunit
JUNB	JunB proto-oncogene, AP-1 transcription factor subunit
LRP1	LDL receptor related protein 1
NECTIN1	nectin cell adhesion molecule 1
NECTIN3	nectin cell adhesion molecule 3
NID2	nidogen 2
NPTX1	neuronal pentraxin 1
NPTX2	neuronal pentraxin 2
NWD2	NACHT and WD repeat domain containing 2
PDIA3	protein disulfide isomerase family A member 3
PRDM16	PR/SET domain 16
RTN4RL1	reticulon 4 receptor like 1
SCUBE1	signal peptide, CUB domain and EGF like domain containing 1
SLC12A7	solute carrier family 12 member 7
SOX4	SRY-box 4
TGFBR2	transforming growth factor beta receptor 2
THBS1	thrombospondin 1
UBC	ubiquitin C
VWF	von Willebrand factor
WFIKKN2	WAP, follistatin/kazal, immunoglobulin, kunitz and netrin domain containing 2

Supplementary Table 2: Genes downregulated by cisplatin and normalized by nasal administration of MSCs

Symbol	Entrez Gene Name
1700048O20Rik	RIKEN cDNA 1700048O20 gene
2810468N07Rik	RIKEN cDNA 2810468N07 gene
ACTN2	actinin alpha 2
ALDH1A1	aldehyde dehydrogenase 1 family member A1
ANLN	anillin actin binding protein
Bhlhe41	basic helix-loop-helix family, member e41
CIRBP	cold inducible RNA binding protein
CPM	carboxypeptidase M
DBP	D-box binding PAR bZIP transcription factor
DLX6	distal-less homeobox 6
EPHA8	EPH receptor A8
EVI2A	ecotropic viral integration site 2A
Fus	fused in sarcoma
GATM	glycine amidinotransferase
GPR83	G protein-coupled receptor 83
HCN2	hyperpolarization activated cyclic nucleotide gated potassium channel 2
IL33	interleukin 33
INSIG1	insulin induced gene 1
KCNAB1	potassium voltage-gated channel subfamily A member regulatory beta subunit 1
LMO7	LIM domain 7
MAG	myelin associated glycoprotein
NKX6-2	NK6 homeobox 2
PLEKHB1	pleckstrin homology domain containing B1
PPP1R14A	protein phosphatase 1 regulatory inhibitor subunit 14A
PRKCH	protein kinase C eta
RAP1GAP	RAP1 GTPase activating protein
RND2	Rho family GTPase 2
S100B	S100 calcium binding protein B
SLC22A3	solute carrier family 22 member 3
TBC1D8	TBC1 domain family member 8
